# Crystal structure of two *N*′-(1-phenyl­benzyl­idene)-2-(thio­phen-3-yl)acetohydrazides

**DOI:** 10.1107/S2056989019008892

**Published:** 2019-07-02

**Authors:** Trung Vu Quoc, Linh Nguyen Ngoc, Duong Tran Thi Thuy, Manh Vu Quoc, Thien Vuong Nguyen, Yen Oanh Doan Thi, Luc Van Meervelt

**Affiliations:** a Faculty of Chemistry, Hanoi National University of Education, 136 Xuan Thuy, Cau Giay, Hanoi, Vietnam; b Bien Hoa Gifted High School, 86 Chu Van An Street, Phu Ly City, Ha Nam Province, Vietnam; c Faculty of Foundation Science, College of Printing Industry, Phuc Dien, Bac Tu Liem, Hanoi, Vietnam; d Institute for Tropical Technology, Vietnam Academy of Science and Technology, 18 Hoang Quoc Viet, Cau Giay, Hanoi, Vietnam; e Graduate University of Science and Technology, Vietnam Academy of Science and Technology, 18 Hoang Quoc Viet, Cau Giay, Hanoi, Vietnam; f Publishing House for Science and Technology, Vietnam Academy of Science and Technology, 18 Hoang Quoc Viet, Cau Giay, Hanoi, Vietnam; g Department of Chemistry, KU Leuven, Biomolecular Architecture, Celestijnenlaan 200F, Leuven (Heverlee), B-3001, Belgium

**Keywords:** crystal structure, acetohydrazides, thio­phene, Hirshfeld analysis

## Abstract

The synthesis, spectroscopic data, crystal and mol­ecular structures of two *N*′-(1-phenyl­benzyl­idene)-2-(thio­phen-3-yl)acetohydrazides, namely *N*′-[1-(4-hy­droxy­phen­yl)benzyl­idene]-2-(thio­phen-3-yl)acetohydrazide, C_13_H_10_N_2_O_2_S, (3a), and *N*′-[1-(4-meth­oxy­phen­yl)benzyl­idene]-2-(thio­phen-3-yl)acetohydrazide, C_14_H_14_N_2_O_2_S, (3b), are described. Both compounds differ in the substituent at the *para* position of the phenyl ring: –OH for (3a) and –OCH_3_ for (3b). In (3a), the thio­phene ring is disordered over two orientations with occupancies of 0.762 (3) and 0.238 (3). The configuration about the C=N bond is *E*. The thio­phene and phenyl rings are inclined by 84.0 (3) and 87.0 (9)° for the major- and minor-occupancy disorder components in (3a), and by 85.89 (12)° in (3b). Although these dihedral angles are similar, the conformation of the linker between the two rings is different [the C—C—C—N torsion angle is −*ac* for (3a) and −*sc* for (3b), while the C6—C7—N9—N10 torsion angle is +*ap* for (3a) and −*sp* for (3b)]. A common feature in the crystal packing of (3a) and (3b) is the presence of N—H⋯O hydrogen bonds, resulting in the formation of chains of mol­ecules running along the *b*-axis direction in the case of (3a), or inversion dimers for (3b). The most prominent contributions to the surface contacts are those in which H atoms are involved, as confirmed by an analysis of the Hirshfeld surface.

## Abstract 2

Two *N*′-(1-(phenyl­ethyl­idene)-2-(thio­phen-3-yl)acetohydrazides containing –OH and –OCH_3_ at the *para*-position of the phenyl ring have been synthesized and their mol­ecular and crystal structures are reported.

## Chemical context   

Acetohydrazides are considered to be good candidates for different pharmaceutical applications, including their use as anti­bacterial, anti­fugal, anti­microbial and anti­convulsant agents (Yadav *et al.*, 2015 ▸; Bharti *et al.*, 2010 ▸; Loncle *et al.*, 2004 ▸; Papakonstanti­nou-Garoufalias *et al.*, 2002 ▸). Moreover, many of them have shown analgesic and anti­platelet properties (Wardakhan *et al.*, 2013 ▸). Combinations of acetohydrazide with other heterocyclic rings have also been investigated, such as the hydrazide-based 2-oxonicotino­nitrile derivatives that are considered to be potential anti­microbial agents (El-Sayed *et al.*, 2018 ▸).

As a continuation of our research (Nguyen *et al.*, 2016 ▸; Vu *et al.*, 2016 ▸, 2017 ▸) on the chemical and physical properties of novel polythio­phenes, a new thio­phene monomer-containing acetohydrazide has been prepared. We have synthesized two *N*′-(1-(phenyl­benzyl­idene)-2-(thio­phen-3-yl)acetohydrazides and present here the spectroscopic data and crystal structures of the title compounds, together with the Hirshfeld surface analysis.
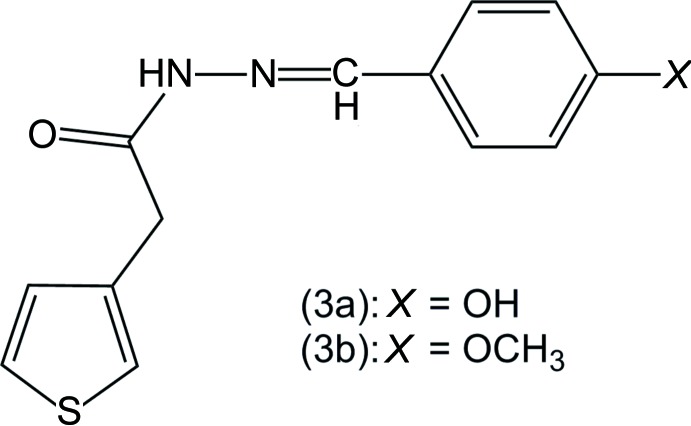


## Structural commentary   

**Figure 1:**
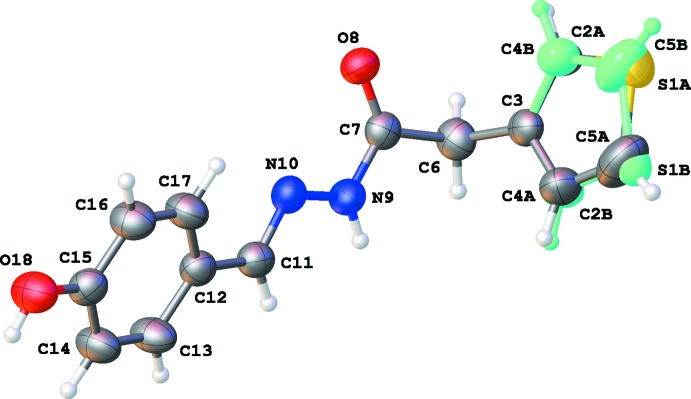
A view of the mol­ecular structure of (3a), with atom labels and displacement ellipsoids drawn at the 50% probability level. The minor-disorder component is shown in light green.

The hy­droxy derivative (3a) crystallizes in the ortho­rhom­bic space group *Pbca*. The thio­phene ring is disordered over two sites (the major and minor components are labelled with the suffixes *A* and *B*, respectively), corresponding to a rotation about the C3—C6 bond of approximately 180° with population parameters 0.762 (3) for S1*A*/C1*A*–C5*A* and 0.238 (3) for S1*B*/C1*B*–C5*B* (Fig. 1 ▸). The configuration of the C11=N10 bond can be described as *E* [the N9—N10—C11—C12 torsion angle is 174.82 (16)°]. The torsion angle C7—N9—N10—C11 of 177.10 (18)° indicates that the conformation around the N9—N10 bond is *+ap*. The mol­ecule is twisted about the C6—C7 bond with a dihedral angle of 84.0 (3)° between the thio­phene and benzene rings [87.0 (9)° for S1*B*/C1*B*–C5*B*] .

**Figure 2:**
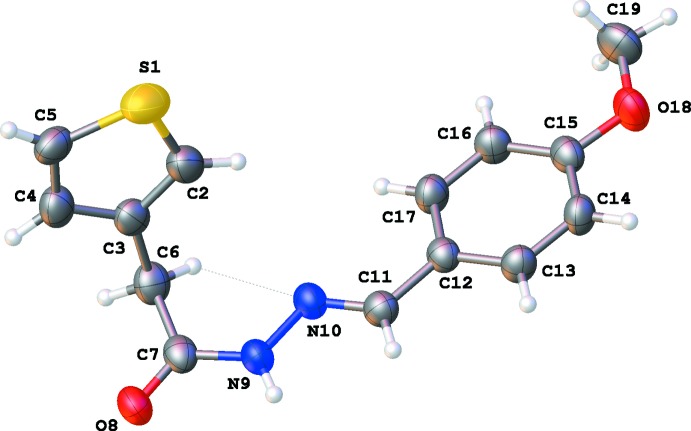
The mol­ecular structure of (3b) with atom labels and 50% probability displacement ellipsoids.

The meth­oxy derivative (3b) (Fig. 2 ▸) crystallizes in the triclinic space group *P* 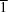. Compared to (3a), the central part of (3b) displays a similar *+ap* conformation around the N9—N10 bond and an *E* configuration of the C11=N10 bond, as illustrated by the torsion angles C7—N9—N10—C11 [177.8 (2)°] and N9—N10—C11—C12 [179.26 (19)°]. However, the conformation about the two other bonds, C6—C7 and especially C7—N9, in the linker between both rings is different. The torsion angle C3—C6—C7—N9 is −101.8 (2)° (or *-ac*) for (3a) and −85.4 (3)° (or *-sc*) for (3b). As a consequence, in (3b) a short C6—H6⋯N10 inter­action occurs (Table 2 ▸). In (3a) we observe an *+ap* conformation [torsion angle C6—C7—N9—N10 is 167.45 (16)°], while this is *-sp* in (3b) [torsion angle C6—C7—N9—N10 is −5.8 (3)°]. The dihedral angle between the thio­phene and phenyl rings is 85.89 (12)°, in the same order as for (3a).

## Supra­molecular features   

**Figure 3:**
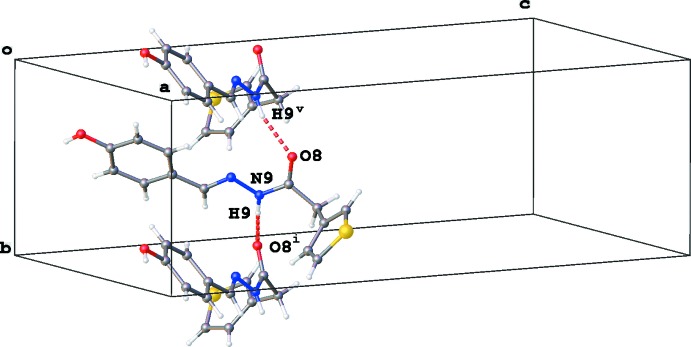
Part of the crystal structure of (3a), showing the chain formation through N—H⋯O inter­actions (red dashed lines) along the *b*-axis direction. The minor disorder component is not shown. Symmetry codes: (i) −*x* + 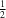, *y* + 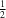, *z;* (v) −*x* + 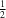, *y* − 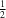, *z.*

**Figure 4:**
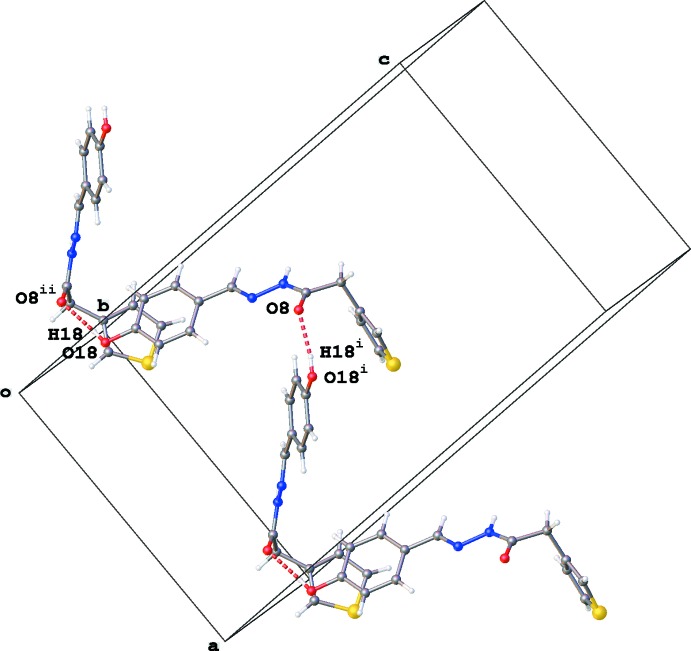
Part of the crystal structure of (3a), illustrating the chain formation through O—H⋯O inter­actions (red dashed lines) along the *a*-axis direction. The minor disorder component is not shown. Symmetry codes: (i) *x* + 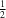, *y*, −*z* + 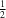 *;* (ii) *x* − 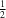, *y*, −*z* + 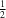.

In the crystal, mol­ecules of (3a) are connected by N9—H9⋯O8^i^ [symmetry code: (i) −*x* + 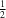, *y* + 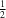, *z*] hydrogen bonds, resulting in the formation of chains in the *b*-axis direction with a 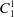
(4) graph-set motif (Fig. 3 ▸, Table 1 ▸). In addition, chains with a 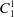
(11) graph-set motif running along the *a*-axis direction are formed by O18—H18⋯O8^ii^ [symmetry code: (ii) *x* − 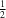, *y*, −*z* + 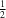] hydrogen bonds (Fig. 4 ▸, Table 1 ▸). Two weaker inter­actions are present in the packing: a C—H⋯O and C—H⋯π(phen­yl) inter­action (for details see Table 1 ▸).

**Figure 5:**
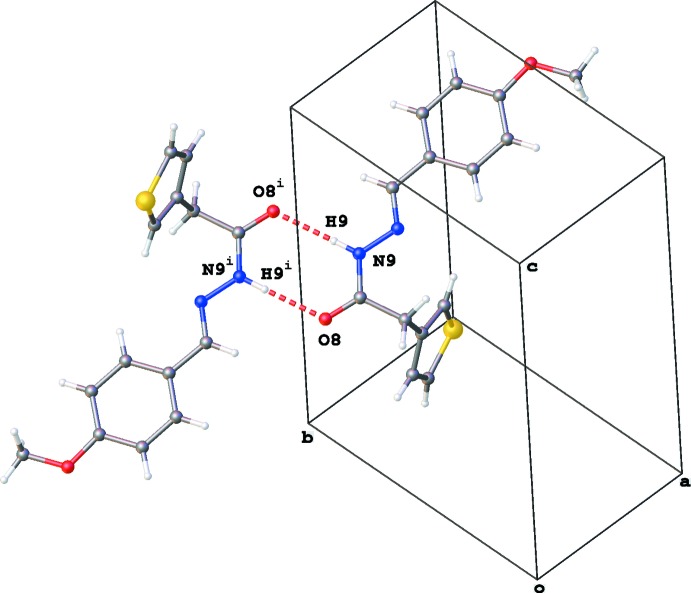
A partial packing diagram of (3b), showing dimer formation through N—H⋯O inter­actions (red dashed lines). Symmetry code: (i) −*x*, −*y* + 2, −*z* + 1.

**Figure 6:**
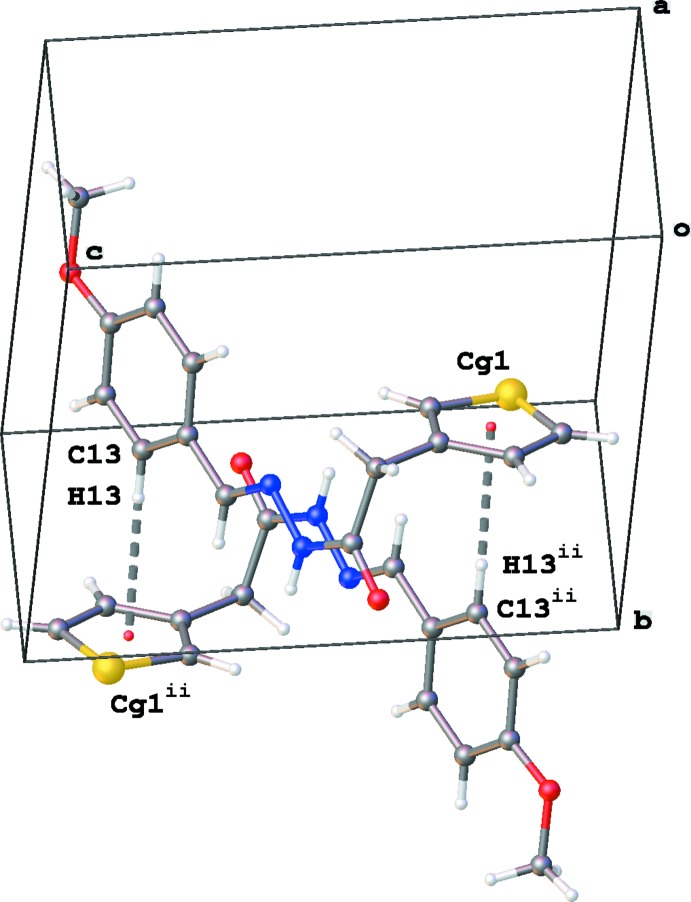
A partial packing diagram of (3b), illustrating the dimer formation through C—H⋯π inter­actions (gray dashed lines). *Cg*1 is the centroid of the S1/C2–C5 thio­phene ring. Symmetry code: (ii) −*x* + 1, −*y* + 2, −*z* + 1.

Replacing the –OH group in (3a) by an –OMe group in (3b) changes the hydrogen-bonding pattern. The crystal packing of (3b) is now characterized by the presence of two different inversion dimers. The first type, with an 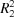
(8) graph-set motif, is formed by N9—H9⋯O8^i^ [symmetry code: (i) −*x*, −*y* + 2, −*z* + 1] hydrogen bonds (Fig. 5 ▸, Table 2 ▸). The second one involves C13—H13⋯π(thio­phene) inter­actions (Fig. 6 ▸, Table 2 ▸).

No voids or π–π stackings are observed in the crystal packing of (3a) and (3b).

## Database survey   

**Figure 7:**
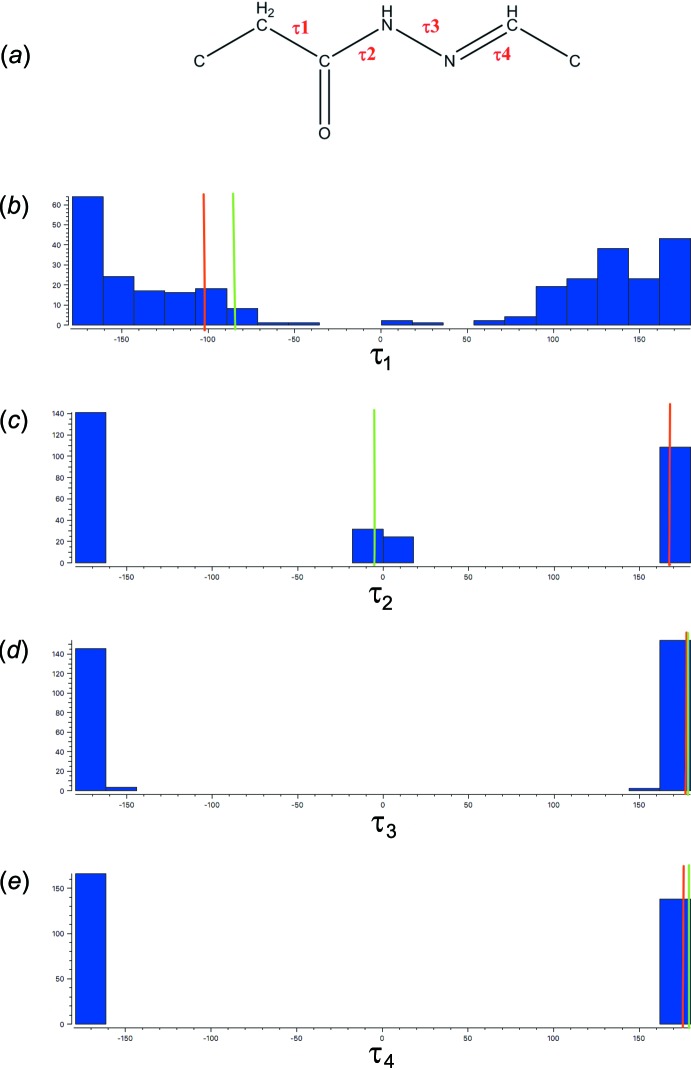
(*a*) Fragment used for a search in the CSD. (*b*)–(*e*) Histograms of torsion angles τ_1_, τ_2_, τ_3_ and τ_4_, respectively. The vertical red and green lines show the torsion angles observed in title compounds (3a) and (3b), respectively.

A search of the Cambridge Structural Database (CSD, Version 5.40, update of May 2019; Groom *et al.*, 2016 ▸) for the central linker between the two rings in the title compound, C—CH_2_—C(=O)—NH—N=CH—C (Fig. 7 ▸ *a*), resulted in 137 hits. Histograms of the distribution of the four torsion angles τ_1_ *–*τ_4_ along the linker backbone are shown in Fig. 7 ▸ *b*–*e* [the red and green lines depict the torsion angles for title compounds (3a) and (3b), respectively]. The histogram of τ_1_ reflects a wide spread with a preference for the −*ap*/+*ap* conformation, followed by the −*sc*/+*sc* conformation and only a few entries in the remaining regions. In the case of torsion angle τ_2_, two regions are preferred: −*ap*/+*ap* [for the majority of the entries and similar to (3a)] and −*sp*/+*sp* [similar to (3b)]. Torsion angles τ_*3*_ and τ_4_ show both a narrow spread in the region −*ap*/+*ap*.

## Hirshfeld surface analysis   

**Figure 8:**
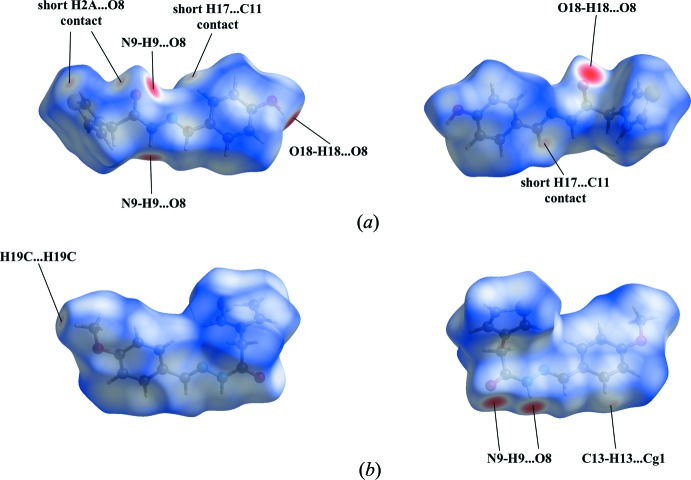
The Hirshfeld surface mapped over *d*_norm_ for (*a*) compound (3a) in the range −0.6166 to 1.1782 a.u., and (*b*) compound (3b) in the range −0.5274 to 1.2642 a.u.

The Hirshfeld surface analysis (Spackman & Jayatilaka, 2009 ▸) and the associated two-dimensional fingerprint plots (McKinnon *et al.*, 2007 ▸) were performed using *CrystalExplorer* (Turner *et al.*, 2017 ▸). The Hirshfeld surfaces of compounds (3a) and (3b) mapped over *d*_norm_ are given in Fig. 8 ▸.

The bright-red spots in Fig. 8 ▸ *a* near atoms O8 and N9 illustrate the N9—H9⋯O8 hydrogen bond, and near atoms O8 and O18 the O18—H18⋯O8 hydrogen bond. The faint-red spots near atoms O8 and H2*A*, and C11 and H17 refer to short contacts in the crystal packing of (3a). The most significant contributions to the Hirshfeld surface are from H⋯H (30.5%), C⋯H/H⋯C (26.1%), O⋯H/H⋯O (18.6%) and S⋯H/H⋯S (10.7%) contacts.

For compound (3b), the N9—H9⋯O8 dimer formation is viewed as the bright-red spots near atoms O8 and N9 in Fig. 8 ▸ *b*. The faint-red spots near atoms H19*C* and H13 are indicative for a short H19*C*⋯H19*C* contact and the C13—H13⋯π(thio­phene) inter­action. The most significant contributions to the Hirshfeld surface are from H⋯H (40.6%), C⋯H/H⋯C (22.2%), O⋯H/H⋯O (15.1%) and S⋯H/H⋯S (12.5%) contacts.

## Synthesis and crystallization   

**Figure 9:**
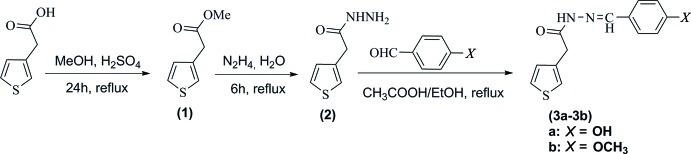
Reaction scheme for the title compounds (3a) and (3b).

The reaction scheme to synthesize the title compounds, (3a) and (3b), is given in Fig. 9 ▸.

Methyl 2-(thio­phen-3-yl)acetate (1) and 2-(thio­phen-3-yl)acetohydrazide (2) were synthesized according to our previous research (Vu *et al.*, 2017 ▸).

***Synthesis of N*** **′** ***-[1-(4-hy­droxy­phen­yl)benzyl­idene]-2-(thio­phen-3-yl)acetohydrazide:***

Compound (2) (3 mmol) and the appropriate benzaldehyde derivatives (6 mmol) with acetic acid (1.5 mL) in ethanol (20 mL) were refluxed for 5 h. The reaction mixture was cooled down and the solid product was separated by filtration and purified by recrystallization in ethanol to give the compounds (3a) and (3b).

***Data for N*** **′** ***-[1-(4-hy­droxy­phen­yl)benzyl­idene]-2-(thio­phen-3-yl)acetohydrazide (3a):***

White crystals; m.p. 443 K; yield 63%. IR (KBr, cm^−1^): 3289, 3207 (NH), 3050, 2874 (C—H), 1621 (C=O), 1606 (CH=N), 1511 (C=C). ^1^H NMR [Bruker XL-500, 500 MHz, *d*_6_-CDCl_3_, δ (ppm), *J* (Hz)]: 7.19 (*m*, 1H, H^2^), 7.11 (*d*, 1H, ^5^ *J* = 5.0, H^4^), 7.25 (*dd*, 1H, ^2^ *J* = 3.0, ^4^ *J* = 5.0, H^5^), 4.07 (*s*, 2H, H^6^), 9.17 (*s*, 1H, H^8^), 7.79 (*s*, 1H, H^9^), 7.52 (*d*, 2H, *J* = 8.5 H^11^, H^15^), 6.87 (*d*, 2H, *J* = 8.5 H^12^, H^14^), 10.10/10.04 (*s*, 1H, H^16^). ^13^C NMR [Bruker XL-500, 125 MHz, *d*_6_-CDCl_3_, δ (ppm)]: 122.3/122.4 (C^2^), 135.3/135.4 (C^3^), 128.7/128.8 (C^4^), 125.4/125.8 (C^5^), 33.6/35.9 (C^6^), 165.7/171.4 (C^7^), 146.7 (C^9^), 143.5 (C^10^), 128.3/128.6 (C^11^, C^15^), 115.6/116.6 (C^12^,C^14^), 159.6/159.2 (C^13^). Calculation for C_13_H_12_N_2_O_2_S: *M* ^[+H]^ = 260.9 au.

***Data for N*** **′** ***-[1-(4-meth­oxy­phen­yl)benzyl­idene]-2-(thio­phen-3-yl)acetohydrazide (3b):***

White crystals, m.p. 431 K, yield 53%. IR (KBr, cm^−1^): 3442, 3112 (NH), 3014, 2950 (C—H), 1706 (C=O), 1617 (CH=N), 1558, 1503 (C=C). ^1^H NMR [Bruker XL-500, 500 MHz, *d*_6_-CDCl_3_, δ (ppm), *J* (Hz)]: 7.22 (*m*, 1H, H^2^); 7.12 (*m*, 1H, H^4^); 7.26 (*dd*, 1H, ^2^ *J* = 3.0, ^5^ *J* = 5.0, H^5^); 4.11 (*s*, 2H, H^6^); 8.97 (*s*, 1H, H^8^); 7.69 (*s*, 1H, H^9^); 7.61 (*d*, 2H, *J* = 8.5, H^11^, H^15^); 6.94 (*d*, 2H, *J* = 8.5, H^12^, H^14^); 3.85 (*m*, 3H, H^16^). ^13^C NMR [Bruker XL-500, 125 MHz, *d*_6_-CDCl_3_, δ (ppm)]: 122.8 (C^2^), 134.4 (C^3^), 129.3 (C^4^), 125.4 (C^5^), 34.3 (C^6^), 172.9 (C^7^), 143.6 (C^9^), 126.4 (C^10^), 128.8 (C^11^, C^15^), 114.3 (C^12^, C^14^), 161.3 (C^13^), 55.4 (C^16^). Calculation for C_14_H_14_N_2_O_2_S: *M* ^[+H]^ = 274.9 au.

## Refinement details   

Crystal data, data collection and structure refinement details are summarized in Table 3 ▸. All H atoms were placed in idealized positions and refined in riding mode, with *U*_iso_(H) values assigned as 1.2*U*_eq_ of the parent atoms (1.5 times for methyl groups), with C—H distances of 0.93 (aromatic), 0.96 (CH_3_) and 0.97 Å (CH_2_), N—H distances of 0.86 Å and O—H distances of 0.82 Å (rotating OH). In (3a), the thio­phene ring is disordered over two positions [population parameters 0.762 (3) and 0.238 (3)] and was refined with restraints for the bond lengths and angles in the ring. The anisotropic temperature factors for atoms S1, C2, C4 and C5 in both orientations were constrained to be equal. In the final cycles of refinement, four and two outliers were omitted for (3a) and (3b), respectively.

## Supplementary Material

Crystal structure: contains datablock(s) 3a, 3b. DOI: 10.1107/S2056989019008892/rz5260sup1.cif

Structure factors: contains datablock(s) 3a. DOI: 10.1107/S2056989019008892/rz52603asup2.hkl

Structure factors: contains datablock(s) 3b. DOI: 10.1107/S2056989019008892/rz52603bsup3.hkl

Supporting information file. DOI: 10.1107/S2056989019008892/rz52603asup4.cml

Supporting information file. DOI: 10.1107/S2056989019008892/rz52603bsup5.cml

CCDC references: 1935593, 1935592

Additional supporting information:  crystallographic information; 3D view; checkCIF report

## Figures and Tables

**Table 1:** Hydrogen-bond geometry (Å, °) for (3a) *Cg*3 is the centroid of the C12–C17 phenyl ring.

*D*—H⋯*A*	*D*—H	H⋯*A*	*D*⋯*A*	*D*—H⋯*A*
N9—H9⋯O8^i^	0.86	2.12	2.953 (2)	162
O18—H18⋯O8^ii^	0.82	1.97	2.782 (2)	169
C2*A*—H2*A*⋯O8^iii^	0.93	2.57	3.439 (7)	155
C13—H13⋯*Cg*3^iv^	0.93	2.89	3.818 (3)	176

Symmetry codes: (i) 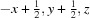; (ii) 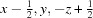; (iii) 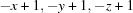; (iv) 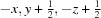.

**Table 2:** Hydrogen-bond geometry (Å, °) for (3b) *Cg*1 is the centroid of the S1/C1–C5 thio­phene ring.

*D*—H⋯*A*	*D*—H	H⋯*A*	*D*⋯*A*	*D*—H⋯*A*
N9—H9⋯O8^i^	0.86	2.08	2.935 (3)	179
C6—H6*A*⋯N10	0.97	2.44	2.782 (3)	100
C13—H13⋯*Cg*1^ii^	0.93	2.68	3.611 (2)	179

Symmetry codes: (i) 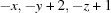; (ii) 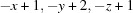.

**Table 3:** Experimental details

	(3a)	(3b)
Crystal data		
Chemical formula	C_13_H_12_N_2_O_2_S	C_14_H_14_N_2_O_2_S
*M*_r_	260.31	274.33
Crystal system, space group	Orthorhombic, *P* *b* *c* *a*	Triclinic, *P* 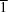
Temperature (K)	293	293
*a*, *b*, *c* (Å)	13.0820 (8), 8.0287 (4), 24.0442 (12)	6.5185 (2), 9.7447 (5), 10.9291 (6)
α, β, γ (°)	90, 90, 90	78.327 (4), 83.070 (4), 87.013 (4)
*V* (Å^3^)	2525.4 (2)	674.63 (6)
*Z*	8	2
Radiation type	Mo *K*α	Mo *K*α
μ (mm^−1^)	0.25	0.24
Crystal size (mm)	0.35 × 0.2 × 0.05	0.5 × 0.15 × 0.05

Data collection		
Diffractometer	Rigaku Oxford Diffraction SuperNova, Single source at offset/far, Eos	Rigaku Oxford Diffraction SuperNova, Single source at offset/far, Eos
Absorption correction	Multi-scan (*CrysAlis PRO*; Rigaku OD, 2018 ▸)	Multi-scan (*CrysAlis PRO*; Rigaku OD, 2018 ▸)
*T*_min_, *T*_max_	0.453, 1.000	0.687, 1.000
No. of measured, independent and observed [*I* > 2σ(*I*)] reflections	13596, 2571, 1759	13795, 2752, 2238
*R*_int_	0.039	0.027
(sin θ/λ)_max_ (Å^−1^)	0.625	0.625

Refinement		
*R*[*F* ^2^ > 2σ(*F* ^2^)], *wR*(*F* ^2^), *S*	0.046, 0.109, 1.07	0.051, 0.145, 1.06
No. of reflections	2571	2752
No. of parameters	178	173
No. of restraints	80	0
H-atom treatment	H-atom parameters constrained	H-atom parameters constrained
Δρ_max_, Δρ_min_ (e Å^−3^)	0.19, −0.18	0.33, −0.38

Computer programs: *CrysAlis PRO* (Rigaku OD, 2018 ▸), *SHELXT* (Sheldrick, 2015 ▸ *a* ▸), *SHELXL* (Sheldrick, 2015 ▸b) and *OLEX2* (Dolomanov *et al.*, 2009 ▸).

## supplementary crystallographic information

### N'-[1-(4-Hydroxyphenyl)benzylidene]-2-(thiophen-3-yl)acetohydrazide (3a) Crystal data

**Table:**

C_13_H_12_N_2_O_2_S	*D*_x_ = 1.369 Mg m^−^^3^
*M**_r_* = 260.31	Mo *K*α radiation, λ = 0.71073 Å
Orthorhombic, *P**b**c**a*	Cell parameters from 3446 reflections
*a* = 13.0820 (8) Å	θ = 3.1–23.7°
*b* = 8.0287 (4) Å	µ = 0.25 mm^−^^1^
*c* = 24.0442 (12) Å	*T* = 293 K
*V* = 2525.4 (2) Å^3^	Plate, white
*Z* = 8	0.35 × 0.2 × 0.05 mm
*F*(000) = 1088	

### N'-[1-(4-Hydroxyphenyl)benzylidene]-2-(thiophen-3-yl)acetohydrazide (3a) Data collection

**Table:**

Rigaku Oxford Diffraction SuperNova, Single source at offset/far, Eos diffractometer	2571 independent reflections
Radiation source: micro-focus sealed X-ray tube, SuperNova (Mo) X-ray Source	1759 reflections with *I* > 2σ(*I*)
Mirror monochromator	*R*_int_ = 0.039
Detector resolution: 15.9631 pixels mm^-1^	θ_max_ = 26.4°, θ_min_ = 3.1°
ω scans	*h* = −16→15
Absorption correction: multi-scan (CrysAlis PRO; Rigaku OD, 2018)	*k* = −9→10
*T*_min_ = 0.453, *T*_max_ = 1.000	*l* = −28→30
13596 measured reflections	

### N'-[1-(4-Hydroxyphenyl)benzylidene]-2-(thiophen-3-yl)acetohydrazide (3a) Refinement

**Table:**

Refinement on *F*^2^	Hydrogen site location: inferred from neighbouring sites
Least-squares matrix: full	H-atom parameters constrained
*R*[*F*^2^ > 2σ(*F*^2^)] = 0.046	*w* = 1/[σ^2^(*F*_o_^2^) + (0.0334*P*)^2^ + 0.6755*P*] where *P* = (*F*_o_^2^ + 2*F*_c_^2^)/3
*wR*(*F*^2^) = 0.109	(Δ/σ)_max_ < 0.001
*S* = 1.07	Δρ_max_ = 0.19 e Å^−^^3^
2571 reflections	Δρ_min_ = −0.18 e Å^−^^3^
178 parameters	Extinction correction: SHELXL (Sheldrick, 2015b), Fc^*^=kFc[1+0.001xFc^2^λ^3^/sin(2θ)]^-1/4^
80 restraints	Extinction coefficient: 0.0022 (6)

### N'-[1-(4-Hydroxyphenyl)benzylidene]-2-(thiophen-3-yl)acetohydrazide (3a) Special details

**Table:**

Geometry. All esds (except the esd in the dihedral angle between two l.s. planes) are estimated using the full covariance matrix. The cell esds are taken into account individually in the estimation of esds in distances, angles and torsion angles; correlations between esds in cell parameters are only used when they are defined by crystal symmetry. An approximate (isotropic) treatment of cell esds is used for estimating esds involving l.s. planes.

### N'-[1-(4-Hydroxyphenyl)benzylidene]-2-(thiophen-3-yl)acetohydrazide (3a) Fractional atomic coordinates and isotropic or equivalent isotropic displacement parameters (Å^2^)

**Table:**

	*x*	*y*	*z*	*U*_iso_*/*U*_eq_	Occ. (<1)
S1A	0.67164 (8)	0.8283 (2)	0.43607 (8)	0.0718 (4)	0.762 (3)
S1B	0.6152 (4)	0.9867 (10)	0.3990 (3)	0.0718 (4)	0.238 (3)
C2A	0.5667 (3)	0.7484 (7)	0.4673 (3)	0.0542 (12)	0.762 (3)
H2A	0.568185	0.659897	0.492216	0.065*	0.762 (3)
C2B	0.4941 (11)	0.970 (3)	0.4213 (16)	0.0542 (12)	0.238 (3)
H2B	0.443026	1.047111	0.413833	0.065*	0.238 (3)
C3	0.47998 (16)	0.8287 (2)	0.45167 (8)	0.0449 (5)	
C4A	0.5018 (5)	0.9582 (10)	0.4141 (5)	0.062 (2)	0.762 (3)
H4A	0.451169	1.026238	0.399205	0.074*	0.762 (3)
C4B	0.5719 (9)	0.741 (3)	0.4587 (11)	0.062 (2)	0.238 (3)
H4B	0.575924	0.640913	0.478166	0.074*	0.238 (3)
C5A	0.6056 (6)	0.9770 (11)	0.4008 (4)	0.100 (3)	0.762 (3)
H5A	0.633233	1.056338	0.376954	0.120*	0.762 (3)
C5B	0.6580 (10)	0.818 (2)	0.4337 (11)	0.100 (3)	0.238 (3)
H5B	0.725438	0.781636	0.435909	0.120*	0.238 (3)
C6	0.37396 (16)	0.7786 (3)	0.47035 (8)	0.0506 (6)	
H6A	0.377254	0.726825	0.506761	0.061*	
H6B	0.330528	0.876200	0.472922	0.061*	
C7	0.33041 (15)	0.6577 (3)	0.42864 (8)	0.0419 (5)	
O8	0.36037 (11)	0.51138 (17)	0.42613 (5)	0.0476 (4)	
N9	0.26256 (13)	0.7198 (2)	0.39247 (6)	0.0455 (4)	
H9	0.236595	0.816955	0.397669	0.055*	
N10	0.23452 (13)	0.6255 (2)	0.34641 (6)	0.0441 (4)	
C11	0.16622 (15)	0.6908 (2)	0.31536 (8)	0.0444 (5)	
H11	0.135667	0.790093	0.326364	0.053*	
C12	0.13478 (16)	0.6139 (2)	0.26302 (8)	0.0428 (5)	
C13	0.04946 (18)	0.6712 (3)	0.23499 (9)	0.0553 (6)	
H13	0.012178	0.759047	0.249952	0.066*	
C14	0.01803 (18)	0.6010 (3)	0.18517 (9)	0.0561 (6)	
H14	−0.040209	0.640454	0.167331	0.067*	
C15	0.07373 (17)	0.4720 (2)	0.16220 (8)	0.0462 (5)	
C16	0.16018 (17)	0.4149 (3)	0.18911 (9)	0.0562 (6)	
H16	0.198233	0.328679	0.173641	0.067*	
C17	0.19021 (17)	0.4850 (3)	0.23870 (9)	0.0538 (6)	
H17	0.248657	0.445441	0.256329	0.065*	
O18	0.04708 (13)	0.39577 (19)	0.11361 (6)	0.0620 (5)	
H18	−0.003809	0.441085	0.100663	0.093*	

### N'-[1-(4-Hydroxyphenyl)benzylidene]-2-(thiophen-3-yl)acetohydrazide (3a) Atomic displacement parameters (Å^2^)

**Table:**

	*U*^11^	*U*^22^	*U*^33^	*U*^12^	*U*^13^	*U*^23^
S1A	0.0493 (6)	0.0807 (8)	0.0853 (7)	0.0003 (5)	−0.0078 (5)	−0.0050 (6)
S1B	0.0493 (6)	0.0807 (8)	0.0853 (7)	0.0003 (5)	−0.0078 (5)	−0.0050 (6)
C2A	0.065 (2)	0.051 (2)	0.047 (3)	0.0092 (16)	−0.0120 (16)	0.0016 (17)
C2B	0.065 (2)	0.051 (2)	0.047 (3)	0.0092 (16)	−0.0120 (16)	0.0016 (17)
C3	0.0513 (14)	0.0403 (11)	0.0430 (11)	−0.0013 (10)	−0.0106 (10)	−0.0057 (9)
C4A	0.060 (3)	0.053 (3)	0.072 (6)	−0.0011 (18)	−0.008 (2)	0.015 (3)
C4B	0.060 (3)	0.053 (3)	0.072 (6)	−0.0011 (18)	−0.008 (2)	0.015 (3)
C5A	0.132 (6)	0.063 (3)	0.103 (4)	−0.025 (3)	−0.011 (3)	0.021 (2)
C5B	0.132 (6)	0.063 (3)	0.103 (4)	−0.025 (3)	−0.011 (3)	0.021 (2)
C6	0.0551 (15)	0.0539 (13)	0.0427 (11)	0.0014 (11)	−0.0020 (10)	−0.0088 (10)
C7	0.0408 (13)	0.0444 (12)	0.0405 (11)	−0.0017 (9)	0.0056 (9)	−0.0002 (9)
O8	0.0533 (10)	0.0400 (8)	0.0495 (8)	0.0012 (7)	−0.0023 (7)	0.0005 (6)
N9	0.0489 (11)	0.0370 (9)	0.0506 (10)	0.0033 (8)	−0.0043 (8)	−0.0084 (8)
N10	0.0471 (11)	0.0408 (10)	0.0444 (9)	−0.0029 (8)	−0.0030 (8)	−0.0029 (8)
C11	0.0451 (13)	0.0420 (11)	0.0461 (11)	0.0020 (9)	0.0006 (10)	0.0014 (9)
C12	0.0458 (13)	0.0404 (11)	0.0424 (11)	−0.0007 (9)	−0.0002 (9)	0.0024 (9)
C13	0.0611 (16)	0.0527 (14)	0.0520 (13)	0.0196 (11)	−0.0082 (11)	−0.0087 (10)
C14	0.0600 (15)	0.0569 (14)	0.0515 (13)	0.0146 (11)	−0.0136 (11)	−0.0038 (11)
C15	0.0553 (14)	0.0429 (12)	0.0405 (11)	0.0003 (10)	−0.0008 (10)	0.0007 (9)
C16	0.0616 (16)	0.0536 (13)	0.0535 (13)	0.0137 (11)	−0.0009 (12)	−0.0088 (11)
C17	0.0516 (15)	0.0545 (13)	0.0553 (13)	0.0117 (11)	−0.0082 (11)	−0.0018 (11)
O18	0.0748 (13)	0.0574 (10)	0.0539 (9)	0.0122 (8)	−0.0116 (8)	−0.0129 (8)

### N'-[1-(4-Hydroxyphenyl)benzylidene]-2-(thiophen-3-yl)acetohydrazide (3a) Geometric parameters (Å, º)

**Table:**

S1A—C2A	1.691 (4)	C7—O8	1.240 (2)
C2A—H2A	0.9300	C7—N9	1.339 (2)
S1B—C2B	1.677 (9)	N9—H9	0.8600
C2B—H2B	0.9300	N9—N10	1.391 (2)
C2A—C3	1.357 (4)	N10—C11	1.277 (2)
C2B—C3	1.360 (9)	C11—H11	0.9300
C4A—H4A	0.9300	C11—C12	1.461 (3)
C4B—H4B	0.9300	C12—C13	1.382 (3)
S1A—C5A	1.700 (7)	C12—C17	1.393 (3)
C4A—C5A	1.404 (6)	C13—H13	0.9300
C5A—H5A	0.9300	C13—C14	1.386 (3)
S1B—C5B	1.688 (9)	C14—H14	0.9300
C4B—C5B	1.419 (9)	C14—C15	1.381 (3)
C5B—H5B	0.9300	C15—C16	1.381 (3)
C3—C4A	1.407 (4)	C15—O18	1.364 (2)
C3—C4B	1.404 (9)	C16—H16	0.9300
C3—C6	1.512 (3)	C16—C17	1.376 (3)
C6—H6A	0.9700	C17—H17	0.9300
C6—H6B	0.9700	O18—H18	0.8200
C6—C7	1.508 (3)		
			
C4A—C5A—S1A	107.6 (5)	C3—C4B—C5B	114.2 (9)
C4B—C5B—S1B	107.2 (8)	O8—C7—C6	121.54 (19)
S1A—C2A—H2A	124.0	O8—C7—N9	122.07 (18)
S1B—C2B—H2B	124.2	N9—C7—C6	116.27 (18)
C5A—C4A—C3	115.0 (5)	C7—N9—H9	120.4
C5A—C4A—H4A	122.5	C7—N9—N10	119.29 (16)
C5B—C4B—H4B	122.9	N10—N9—H9	120.4
C2A—S1A—C5A	94.3 (3)	C11—N10—N9	115.25 (17)
S1A—C5A—H5A	126.2	N10—C11—H11	119.1
C4A—C5A—H5A	126.2	N10—C11—C12	121.81 (19)
C2B—S1B—C5B	95.2 (6)	C12—C11—H11	119.1
S1B—C5B—H5B	126.4	C13—C12—C11	120.45 (18)
C4B—C5B—H5B	126.4	C13—C12—C17	117.57 (19)
C2A—C3—C4A	111.1 (3)	C17—C12—C11	121.95 (19)
C2B—C3—C4B	111.5 (7)	C12—C13—H13	119.1
C4A—C3—C6	125.0 (3)	C12—C13—C14	121.7 (2)
C2A—C3—C6	123.9 (3)	C14—C13—H13	119.1
C4B—C3—C6	128.0 (6)	C13—C14—H14	120.2
C2B—C3—C6	120.4 (5)	C15—C14—C13	119.6 (2)
C3—C6—H6A	110.0	C15—C14—H14	120.2
C3—C2A—S1A	112.1 (3)	C14—C15—C16	119.6 (2)
C3—C2B—S1B	111.6 (7)	O18—C15—C14	123.0 (2)
C3—C6—H6B	110.0	O18—C15—C16	117.47 (19)
H6A—C6—H6B	108.3	C15—C16—H16	119.9
C3—C2A—H2A	124.0	C17—C16—C15	120.3 (2)
C3—C2B—H2B	124.2	C17—C16—H16	119.9
C7—C6—C3	108.68 (16)	C12—C17—H17	119.4
C7—C6—H6A	110.0	C16—C17—C12	121.3 (2)
C3—C4A—H4A	122.5	C16—C17—H17	119.4
C3—C4B—H4B	122.9	C15—O18—H18	109.5
C7—C6—H6B	110.0		
			
C2A—S1A—C5A—C4A	−0.7 (11)	C6—C3—C4B—C5B	176.8 (17)
C2B—S1B—C5B—C4B	5 (3)	C6—C3—C4A—C5A	−177.0 (8)
C5B—S1B—C2B—C3	−5 (3)	C6—C7—N9—N10	167.45 (16)
C5A—S1A—C2A—C3	0.8 (7)	C7—N9—N10—C11	177.10 (18)
S1B—C2B—C3—C4B	3 (3)	O8—C7—N9—N10	−8.6 (3)
S1A—C2A—C3—C4A	−0.7 (5)	N9—N10—C11—C12	174.82 (16)
S1A—C2A—C3—C6	176.5 (3)	N10—C11—C12—C13	169.1 (2)
S1B—C2B—C3—C6	−173.1 (14)	N10—C11—C12—C17	−12.8 (3)
C2B—C3—C6—C7	95 (2)	C11—C12—C13—C14	179.8 (2)
C2A—C3—C6—C7	−91.1 (5)	C11—C12—C17—C16	−179.4 (2)
C4A—C3—C6—C7	85.7 (7)	C12—C13—C14—C15	−0.9 (4)
C4B—C3—C6—C7	−81.0 (17)	C13—C12—C17—C16	−1.2 (3)
C2A—C3—C4A—C5A	0.1 (11)	C13—C14—C15—C16	−0.1 (3)
C2B—C3—C4B—C5B	1 (3)	C13—C14—C15—O18	179.4 (2)
C3—C4A—C5A—S1A	0.5 (14)	C14—C15—C16—C17	0.5 (3)
C3—C4B—C5B—S1B	−4 (3)	C15—C16—C17—C12	0.1 (3)
C3—C6—C7—O8	74.2 (2)	C17—C12—C13—C14	1.6 (3)
C3—C6—C7—N9	−101.8 (2)	O18—C15—C16—C17	−179.0 (2)

### N'-[1-(4-Hydroxyphenyl)benzylidene]-2-(thiophen-3-yl)acetohydrazide (3a) Hydrogen-bond geometry (Å, º)

*Cg*3 is the centroid of the C12–C17 phenyl ring.

**Table:**

*D*—H···*A*	*D*—H	H···*A*	*D*···*A*	*D*—H···*A*
N9—H9···O8^i^	0.86	2.12	2.953 (2)	162
O18—H18···O8^ii^	0.82	1.97	2.782 (2)	169
C2*A*—H2*A*···O8^iii^	0.93	2.57	3.439 (7)	155
C13—H13···*Cg*3^iv^	0.93	2.89	3.818 (3)	176

Symmetry codes: (i) −*x*+1/2, *y*+1/2, *z*; (ii) *x*−1/2, *y*, −*z*+1/2; (iii) −*x*+1, −*y*+1, −*z*+1; (iv) −*x*, *y*+1/2, −*z*+1/2.

### N'-[1-(4-Methoxyphenyl)benzylidene]-2-(thiophen-3-yl)acetohydrazide (3b) Crystal data

**Table:**

C_14_H_14_N_2_O_2_S	*Z* = 2
*M**_r_* = 274.33	*F*(000) = 288
Triclinic, *P*1	*D*_x_ = 1.350 Mg m^−^^3^
*a* = 6.5185 (2) Å	Mo *K*α radiation, λ = 0.71073 Å
*b* = 9.7447 (5) Å	Cell parameters from 5534 reflections
*c* = 10.9291 (6) Å	θ = 3.1–27.2°
α = 78.327 (4)°	µ = 0.24 mm^−^^1^
β = 83.070 (4)°	*T* = 293 K
γ = 87.013 (4)°	Needle, white
*V* = 674.63 (6) Å^3^	0.5 × 0.15 × 0.05 mm

### N'-[1-(4-Methoxyphenyl)benzylidene]-2-(thiophen-3-yl)acetohydrazide (3b) Data collection

**Table:**

Rigaku Oxford Diffraction SuperNova, Single source at offset/far, Eos diffractometer	2752 independent reflections
Radiation source: micro-focus sealed X-ray tube, SuperNova (Mo) X-ray Source	2238 reflections with *I* > 2σ(*I*)
Mirror monochromator	*R*_int_ = 0.027
Detector resolution: 15.9631 pixels mm^-1^	θ_max_ = 26.4°, θ_min_ = 2.6°
ω scans	*h* = −8→8
Absorption correction: multi-scan (CrysAlis PRO; Rigaku OD, 2018)	*k* = −12→12
*T*_min_ = 0.687, *T*_max_ = 1.000	*l* = −13→13
13795 measured reflections	

### N'-[1-(4-Methoxyphenyl)benzylidene]-2-(thiophen-3-yl)acetohydrazide (3b) Refinement

**Table:**

Refinement on *F*^2^	0 restraints
Least-squares matrix: full	Hydrogen site location: inferred from neighbouring sites
*R*[*F*^2^ > 2σ(*F*^2^)] = 0.051	H-atom parameters constrained
*wR*(*F*^2^) = 0.145	*w* = 1/[σ^2^(*F*_o_^2^) + (0.0537*P*)^2^ + 0.5294*P*] where *P* = (*F*_o_^2^ + 2*F*_c_^2^)/3
*S* = 1.06	(Δ/σ)_max_ < 0.001
2752 reflections	Δρ_max_ = 0.33 e Å^−^^3^
173 parameters	Δρ_min_ = −0.38 e Å^−^^3^

### N'-[1-(4-Methoxyphenyl)benzylidene]-2-(thiophen-3-yl)acetohydrazide (3b) Special details

**Table:**

Geometry. All esds (except the esd in the dihedral angle between two l.s. planes) are estimated using the full covariance matrix. The cell esds are taken into account individually in the estimation of esds in distances, angles and torsion angles; correlations between esds in cell parameters are only used when they are defined by crystal symmetry. An approximate (isotropic) treatment of cell esds is used for estimating esds involving l.s. planes.

### N'-[1-(4-Methoxyphenyl)benzylidene]-2-(thiophen-3-yl)acetohydrazide (3b) Fractional atomic coordinates and isotropic or equivalent isotropic displacement parameters (Å^2^)

**Table:**

	*x*	*y*	*z*	*U*_iso_*/*U*_eq_	
S1	0.68895 (12)	0.78924 (10)	0.16957 (9)	0.0734 (3)	
C2	0.5595 (4)	0.7356 (3)	0.3147 (3)	0.0534 (6)	
H2	0.619153	0.726965	0.389168	0.064*	
C3	0.3579 (4)	0.7066 (2)	0.3100 (2)	0.0436 (5)	
C4	0.3123 (4)	0.7275 (3)	0.1841 (2)	0.0512 (6)	
H4	0.182310	0.712513	0.163071	0.061*	
C5	0.4790 (4)	0.7727 (3)	0.0935 (2)	0.0497 (6)	
H5	0.476097	0.790729	0.006852	0.060*	
C6	0.2002 (4)	0.6708 (3)	0.4234 (2)	0.0479 (6)	
H6A	0.265853	0.615910	0.493142	0.057*	
H6B	0.093072	0.615347	0.404620	0.057*	
C7	0.1056 (3)	0.8042 (3)	0.4589 (2)	0.0418 (5)	
O8	−0.0513 (2)	0.86009 (19)	0.41630 (16)	0.0508 (4)	
N9	0.2051 (3)	0.8658 (2)	0.53376 (18)	0.0417 (5)	
H9	0.160089	0.945717	0.549350	0.050*	
N10	0.3778 (3)	0.8031 (2)	0.58594 (17)	0.0412 (5)	
C11	0.4621 (3)	0.8739 (2)	0.6517 (2)	0.0411 (5)	
H11	0.406000	0.961642	0.660165	0.049*	
C12	0.6438 (3)	0.8207 (2)	0.7139 (2)	0.0372 (5)	
C13	0.7354 (4)	0.9057 (2)	0.7794 (2)	0.0423 (5)	
H13	0.679005	0.994741	0.782354	0.051*	
C14	0.9070 (4)	0.8605 (2)	0.8396 (2)	0.0444 (5)	
H14	0.966061	0.919004	0.882266	0.053*	
C15	0.9921 (3)	0.7276 (2)	0.8368 (2)	0.0409 (5)	
C16	0.9037 (4)	0.6415 (2)	0.7722 (2)	0.0442 (5)	
H16	0.960075	0.552276	0.769944	0.053*	
C17	0.7322 (4)	0.6880 (2)	0.7113 (2)	0.0435 (5)	
H17	0.674636	0.629647	0.667808	0.052*	
O18	1.1602 (3)	0.69079 (19)	0.90073 (17)	0.0565 (5)	
C19	1.2603 (4)	0.5592 (3)	0.8926 (3)	0.0634 (8)	
H19A	1.307244	0.557208	0.806228	0.095*	
H19B	1.164788	0.485433	0.925510	0.095*	
H19C	1.376398	0.546115	0.940466	0.095*	

### N'-[1-(4-Methoxyphenyl)benzylidene]-2-(thiophen-3-yl)acetohydrazide (3b) Atomic displacement parameters (Å^2^)

**Table:**

	*U*^11^	*U*^22^	*U*^33^	*U*^12^	*U*^13^	*U*^23^
S1	0.0554 (5)	0.0848 (6)	0.0861 (6)	−0.0076 (4)	0.0084 (4)	−0.0394 (5)
C2	0.0441 (13)	0.0592 (16)	0.0640 (16)	0.0019 (11)	−0.0103 (12)	−0.0270 (13)
C3	0.0445 (13)	0.0376 (12)	0.0529 (14)	−0.0006 (9)	−0.0085 (10)	−0.0175 (10)
C4	0.0541 (15)	0.0493 (14)	0.0568 (15)	−0.0023 (11)	−0.0156 (12)	−0.0207 (12)
C5	0.0528 (14)	0.0505 (14)	0.0506 (14)	−0.0044 (11)	0.0003 (11)	−0.0238 (11)
C6	0.0503 (14)	0.0436 (13)	0.0526 (14)	−0.0098 (10)	−0.0110 (11)	−0.0108 (11)
C7	0.0368 (12)	0.0511 (13)	0.0368 (11)	−0.0088 (10)	−0.0037 (9)	−0.0056 (10)
O8	0.0366 (9)	0.0680 (12)	0.0511 (10)	−0.0008 (8)	−0.0129 (7)	−0.0152 (8)
N9	0.0364 (10)	0.0489 (11)	0.0424 (10)	0.0032 (8)	−0.0114 (8)	−0.0121 (8)
N10	0.0381 (10)	0.0472 (11)	0.0386 (10)	0.0011 (8)	−0.0096 (8)	−0.0069 (8)
C11	0.0421 (12)	0.0422 (12)	0.0396 (12)	0.0021 (9)	−0.0073 (9)	−0.0087 (9)
C12	0.0370 (11)	0.0399 (12)	0.0343 (11)	−0.0020 (9)	−0.0055 (9)	−0.0052 (9)
C13	0.0485 (13)	0.0361 (12)	0.0436 (12)	0.0034 (10)	−0.0101 (10)	−0.0098 (9)
C14	0.0498 (13)	0.0439 (13)	0.0444 (13)	−0.0029 (10)	−0.0149 (10)	−0.0143 (10)
C15	0.0393 (12)	0.0467 (13)	0.0356 (11)	−0.0006 (10)	−0.0088 (9)	−0.0034 (9)
C16	0.0482 (13)	0.0365 (12)	0.0491 (13)	0.0036 (10)	−0.0117 (11)	−0.0094 (10)
C17	0.0470 (13)	0.0408 (12)	0.0465 (13)	−0.0032 (10)	−0.0113 (10)	−0.0135 (10)
O18	0.0538 (10)	0.0595 (11)	0.0613 (11)	0.0109 (8)	−0.0284 (9)	−0.0142 (9)
C19	0.0560 (16)	0.0645 (18)	0.0690 (18)	0.0175 (13)	−0.0214 (14)	−0.0085 (14)

### N'-[1-(4-Methoxyphenyl)benzylidene]-2-(thiophen-3-yl)acetohydrazide (3b) Geometric parameters (Å, º)

**Table:**

S1—C2	1.700 (3)	C11—H11	0.9300
S1—C5	1.715 (3)	C11—C12	1.456 (3)
C2—H2	0.9300	C12—C13	1.396 (3)
C2—C3	1.368 (3)	C12—C17	1.393 (3)
C3—C4	1.415 (3)	C13—H13	0.9300
C3—C6	1.507 (3)	C13—C14	1.374 (3)
C4—H4	0.9300	C14—H14	0.9300
C4—C5	1.403 (4)	C14—C15	1.387 (3)
C5—H5	0.9300	C15—C16	1.387 (3)
C6—H6A	0.9700	C15—O18	1.363 (3)
C6—H6B	0.9700	C16—H16	0.9300
C6—C7	1.511 (3)	C16—C17	1.379 (3)
C7—O8	1.230 (3)	C17—H17	0.9300
C7—N9	1.348 (3)	O18—C19	1.422 (3)
N9—H9	0.8600	C19—H19A	0.9600
N9—N10	1.382 (2)	C19—H19B	0.9600
N10—C11	1.277 (3)	C19—H19C	0.9600
			
C2—S1—C5	93.48 (13)	N10—C11—C12	121.7 (2)
S1—C2—H2	123.7	C12—C11—H11	119.2
C3—C2—S1	112.5 (2)	C13—C12—C11	119.1 (2)
C3—C2—H2	123.7	C17—C12—C11	123.1 (2)
C2—C3—C4	111.0 (2)	C17—C12—C13	117.8 (2)
C2—C3—C6	124.5 (2)	C12—C13—H13	119.3
C4—C3—C6	124.3 (2)	C14—C13—C12	121.4 (2)
C3—C4—H4	122.7	C14—C13—H13	119.3
C5—C4—C3	114.5 (2)	C13—C14—H14	120.0
C5—C4—H4	122.7	C13—C14—C15	120.0 (2)
S1—C5—H5	125.7	C15—C14—H14	120.0
C4—C5—S1	108.50 (19)	C14—C15—C16	119.5 (2)
C4—C5—H5	125.7	O18—C15—C14	116.0 (2)
C3—C6—H6A	109.8	O18—C15—C16	124.5 (2)
C3—C6—H6B	109.8	C15—C16—H16	119.9
C3—C6—C7	109.57 (19)	C17—C16—C15	120.1 (2)
H6A—C6—H6B	108.2	C17—C16—H16	119.9
C7—C6—H6A	109.8	C12—C17—H17	119.4
C7—C6—H6B	109.8	C16—C17—C12	121.2 (2)
O8—C7—C6	121.7 (2)	C16—C17—H17	119.4
O8—C7—N9	120.2 (2)	C15—O18—C19	117.5 (2)
N9—C7—C6	117.9 (2)	O18—C19—H19A	109.5
C7—N9—H9	119.3	O18—C19—H19B	109.5
C7—N9—N10	121.3 (2)	O18—C19—H19C	109.5
N10—N9—H9	119.3	H19A—C19—H19B	109.5
C11—N10—N9	115.4 (2)	H19A—C19—H19C	109.5
N10—C11—H11	119.2	H19B—C19—H19C	109.5
			
S1—C2—C3—C4	0.8 (3)	N10—C11—C12—C13	176.9 (2)
S1—C2—C3—C6	−173.66 (19)	N10—C11—C12—C17	−3.1 (4)
C2—S1—C5—C4	0.8 (2)	C11—C12—C13—C14	180.0 (2)
C2—C3—C4—C5	−0.2 (3)	C11—C12—C17—C16	−179.6 (2)
C2—C3—C6—C7	84.9 (3)	C12—C13—C14—C15	−0.4 (4)
C3—C4—C5—S1	−0.5 (3)	C13—C12—C17—C16	0.4 (3)
C3—C6—C7—O8	90.8 (3)	C13—C14—C15—C16	0.4 (4)
C3—C6—C7—N9	−85.4 (3)	C13—C14—C15—O18	−179.0 (2)
C4—C3—C6—C7	−88.8 (3)	C14—C15—C16—C17	0.0 (4)
C5—S1—C2—C3	−1.0 (2)	C14—C15—O18—C19	−175.7 (2)
C6—C3—C4—C5	174.3 (2)	C15—C16—C17—C12	−0.4 (4)
C6—C7—N9—N10	−5.8 (3)	C16—C15—O18—C19	4.9 (4)
C7—N9—N10—C11	177.8 (2)	C17—C12—C13—C14	0.0 (3)
O8—C7—N9—N10	177.97 (19)	O18—C15—C16—C17	179.4 (2)
N9—N10—C11—C12	179.26 (19)		

### N'-[1-(4-Methoxyphenyl)benzylidene]-2-(thiophen-3-yl)acetohydrazide (3b) Hydrogen-bond geometry (Å, º)

*Cg*1 is the centroid of the S1/C1–C5 thiophene ring.

**Table:**

*D*—H···*A*	*D*—H	H···*A*	*D*···*A*	*D*—H···*A*
N9—H9···O8^i^	0.86	2.08	2.935 (3)	179
C6—H6*A*···N10	0.97	2.44	2.782 (3)	100
C13—H13···*Cg*1^ii^	0.93	2.68	3.611 (2)	179

Symmetry codes: (i) −*x*, −*y*+2, −*z*+1; (ii) −*x*+1, −*y*+2, −*z*+1.
